# Fracture Strength of Implant-Supported Hybrid Abutment Crowns: An In Vitro Study of Ceramic and Polymer-Based Materials in the Premolar Region

**DOI:** 10.3390/jcm14238525

**Published:** 2025-12-01

**Authors:** Derya Arısan, Ender Kazazoğlu

**Affiliations:** 1 Independent Researcher, Ankara 06000, Turkey; 2 Department of Prosthodontics, Faculty of Dentistry, Yeditepe University, Istanbul 34755, Turkey; ekazazoglu@hotmail.com

**Keywords:** hybrid abutment, fracture resistance, Ti-base abutment, implant-supported crown, CAD/CAM implant restoration

## Abstract

**Background/Objectives**: Ceramic and polymer-based abutments and crowns are increasingly used for esthetic implant restorations, but their mechanical reliability under functional loading remains unclear. This study aimed to evaluate the fracture strength of implant-supported restorations with different abutment–crown material combinations. **Methods**: Ninety titanium implants (4.1 × 15 mm; BEGO, Germany) were restored with nine combinations of CAD/CAM-fabricated abutment and crown materials (zirconia, lithium disilicate, and ceramic-reinforced polymer; crowns of zirconia, advanced lithium disilicate, and hybrid nanoceramic; *n* = 10 per group). Ti-base abutments were bonded and cemented following material-specific surface treatments and thermocycled 5000 times (5–55 °C). Fracture tests were performed under static vertical loading at 1 mm/min in a universal testing machine. Data were analyzed using two-way ANOVA and Tukey HSD (α = 0.05). **Results**: Fracture resistance differed significantly among groups (*p* < 0.001). The highest mean strength was obtained for zirconia abutment–zirconia crown restorations (1417 N), followed by lithium-disilicate abutment–zirconia crown (1349 N), whereas BioHPP abutment–Tessera crown showed the lowest (823 N). Hybrid composite (Cerasmart) crowns exhibited stable performance across abutments, while Tessera crowns showed lower resistance. BioHPP abutments produced only crown-level fractures (*p* = 0.004), indicating a more reparable failure mode. **Conclusions**: Zirconia-based combinations showed the highest fracture resistance and are suitable for posterior use. Clinicians should balance strength with esthetics when considering translucent materials like advanced lithium disilicate or hybrid ceramics. Long-term clinical studies are needed to confirm these results and guide material selection.

## 1. Introduction

Achieving long-term success in implant dentistry relies on both mechanical stability and natural esthetics. Single-tooth restorations in the anterior and premolar regions present unique challenges, particularly in patients with high smile lines and thin gingival biotypes, which can compromise esthetic outcomes [1,2]. Achieving optimal esthetic and functional outcomes in implant-supported restorations, particularly in the premolar and posterior regions, requires careful management of peri-implant soft tissue contours, implant positioning, and abutment selection, as emphasized in recent studies on soft-tissue stability and pink esthetic restorations [3,4].

Titanium abutments are widely preferred in implant dentistry because of their proven compatibility with biological tissues and high mechanical resilience; however, their metallic appearance may negatively affect visual outcomes, especially in patients with thin gingival tissues. In posterior regions, recent systematic reviews have reported that alternative abutment materials can achieve comparable mechanical reliability with improved optical outcomes [5,6]. To address this limitation, alternative materials such as zirconia, lithium disilicate, and high-performance polymers have been introduced to enhance translucency and color stability [7,8,9].

Hybrid abutment crowns (HACs) integrate a standardized titanium insert with an externally bonded restoration made from ceramic or polymer materials, aiming to unify mechanical strength with esthetic appeal [10,11,12]. HACs can be fabricated as one-piece (monoblock) restorations or as two-piece designs, where the crown is cemented over the bonded abutment. One-piece configurations streamline fabrication and reduce the risk of misfit [12]. Tooth-colored CAD/CAM materials commonly used in HACs include 3Y-TZP zirconia, lithium disilicate, and reinforced polymer-based materials. While 3Y zirconia is known for its strength but limited translucency, the 4Y and 5Y formulations offer enhanced esthetics along with moderate durability [13]. Lithium disilicate is preferred for its translucency and color integration, making it a viable option in esthetically visible posterior regions, such as premolars in patients with high smile lines [14]. BioHPP, a ceramic-reinforced PEEK, offers a lightweight, elastic, and metal-free alternative suitable for patients with metal sensitivities. Hybrid ceramics combine ceramic rigidity with resin flexibility, enhancing machinability and resistance to chipping, although their lower hardness may limit their use in high-load areas [15,16]. Despite these advances, clinical data on the long-term performance of polymer-based abutments and hybrid ceramics in HACs remain limited.

To simulate intraoral aging, thermal cycling was applied based on ISO/TS 11405:2015 [17] protocols, which replicate the chemical and mechanical fatigue experienced under clinical conditions [18]. Elsayed et al. further demonstrated superior fatigue resistance of zirconia abutments compared to lithium disilicate, supporting the rationale for material-specific testing [19].

Considering the increasing use of hybrid abutments for implant-supported prostheses driven by rapid advancements in digital fabrication technologies, clinicians require fast and reliable in vitro data until long-term clinical studies become available. Although various CAD/CAM materials, including zirconia, lithium disilicate, and ceramic-reinforced polymer, are currently used to fabricate hybrid abutments on titanium bases, the available evidence regarding their mechanical performance remains limited. Most existing studies evaluate these materials separately or under differing abutment designs and testing conditions, making direct comparison difficult.

Therefore, the aim of this in vitro study was to evaluate the effect of abutment material (zirconia, lithium disilicate, and BioHPP) and crown material (zirconia, lithium disilicate, and hybrid ceramic) on the fracture strength and failure modes of implant-supported hybrid abutment crowns. The null hypothesis stated that neither the abutment material nor the crown material, nor their interaction, would significantly affect the fracture strength of the tested restorations.

## 2. Materials and Methods

### 2.1. Study Design and Experimental Groups

**Figure 1:**
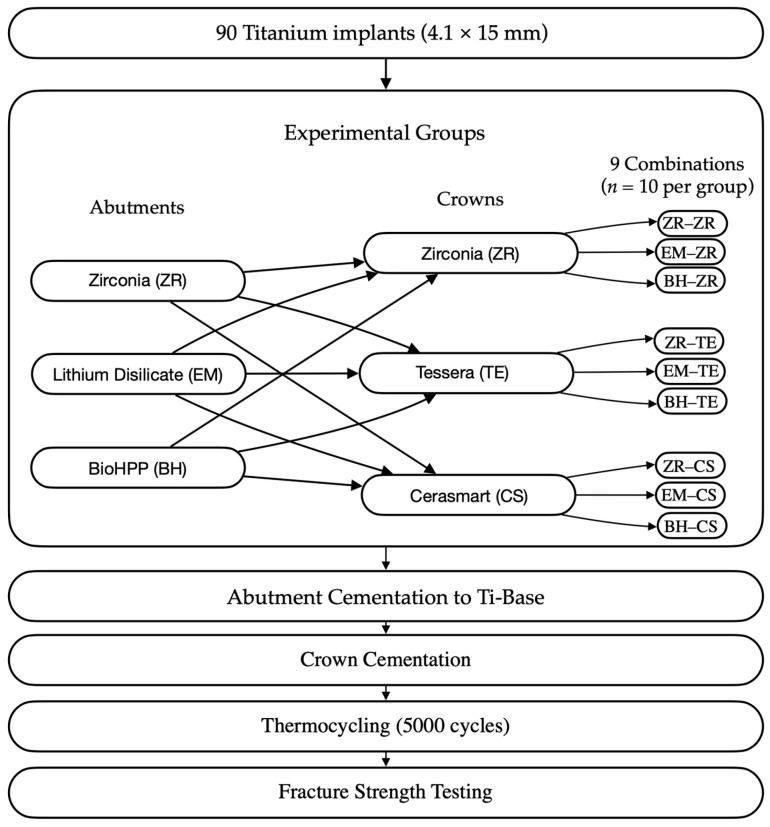
Schematic workflow of the study protocol.

**Figure 2:**
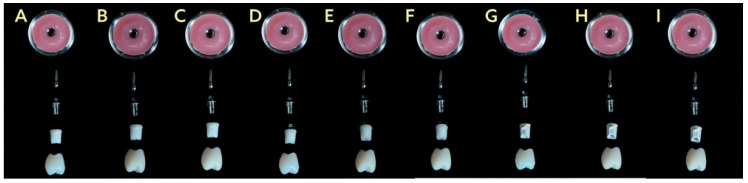
Representative specimens from the nine experimental groups (*n* = 10 each) illustrating different abutment–crown material combinations (**A**–**I**).

This in vitro study evaluated the fracture resistance of implant-supported restorations using nine different combinations of CAD/CAM-fabricated abutment and crown materials (Figure 1). Three types of monolithic materials were used for abutments: 5Y-TZP zirconia (DD CubeX^2^; Dental Direkt GmbH, Spenge, Germany) (ZR), lithium disilicate (IPS e.max CAD; Ivoclar Vivadent AG, Schaan, Liechtenstein) (EM), and a ceramic-reinforced polymer (BreCAM.BioHPP; Bredent GmbH & Co. KG, Senden, Germany) (BioHPP, BH). Crowns were also fabricated from three different monolithic CAD/CAM materials: 5Y-TZP zirconia (DD CubeX^2^; Dental Direkt GmbH, Spenge, Germany) (ZR), advanced lithium disilicate (CEREC Tessera; Dentsply Sirona, Charlotte, NC, USA) (Tessera, TE), and a hybrid nanoceramic (GC Cerasmart; GC Corporation, Tokyo, Japan) (GC Cerasmart, CS). The IPS e.max CAD abutments consisted of a lithium-disilicate glass-ceramic with Li_2_Si_2_O_5_ crystals dispersed in a silica-based matrix. BioHPP abutments were made from a PEEK polymer reinforced with ceramic microfillers. Zirconia abutments were fabricated from 5Y-TZP containing zirconium dioxide stabilized with 5 mol% yttria. The crown materials included the same 5Y-TZP zirconia, Tessera advanced glass-ceramic composed of lithium-disilicate and virgilite crystals, and CeraSmart, a nanoceramic hybrid resin block with approximately 70 wt% silica and barium-glass fillers. In total, 90 specimens were fabricated to simulate single-unit restorations in the maxillary first premolar region. All groups, including their respective combinations of abutment and crown materials, are summarized in Table 1, and representative images are shown in Figure 2.

### 2.2. Implant Selection and Positioning

A total of 90 titanium implants (4.1 × 15 mm; BEGO GmbH & Co. KG, Bremen, Germany) and prefabricated titanium base abutments (RSX Ti-bases, BEGO GmbH & Co. KG, Bremen, Germany) were used to simulate the restoration of a maxillary first premolar. Custom metal molds (4.1 mm in diameter, 16 mm in length) compatible with the implant dimensions were fabricated to ensure standardized cementation and stabilization. A supporting mold was used to stabilize the specimens during embedding. All implants were embedded in autopolymerizing acrylic resin using a parallelometer (Paraskop M; BEGO GmbH & Co. KG, Bremen, Germany) to ensure precise vertical alignment, leaving approximately 1 mm of the implant neck exposed to simulate bone-level support. After abutment fabrication and cementation, each crown was seated with gentle uniform pressure to ensure complete adaptation, and correct positioning was verified visually. During fracture testing, each implant–abutment–crown assembly was secured in a stainless-steel holder compatible with the universal testing machine (Instron 3345, Instron Corp., Norwood, MA, USA) to ensure uniform rigidity and reproducible load distribution.

Sample size was determined using G*Power software (Version 3.1.9.7, Heinrich-Heine-Universität Düsseldorf, Düsseldorf, Germany) for a one-way ANOVA with nine independent groups, assuming an alpha (α) level of 0.05 and a desired power (1 − β) of 80%. Based on a review of similar in vitro studies investigating the fracture resistance of CAD/CAM implant-supported restorations with hybrid abutment designs [20,21], which reported substantial differences in fracture strength between material combinations, a large effect size (f = 0.43) was assumed. The power analysis indicated that 10 specimens per group would be sufficient to detect statistically significant differences, resulting in a total sample size of 90 specimens. This sample size is consistent with established methodologies in comparable research on hybrid abutment crowns [21,22]. It should be noted that the power calculation was based on a large effect size, meaning smaller intergroup differences may not have been detected.

### 2.3. Abutment Fabrication

To standardize abutments, a maxillary model with an edentulous first premolar site was used. An implant analog was placed, and a provisional tooth was positioned to verify proximal contacts. A scan body was mounted directly onto the implant analog and scanned using an intraoral scanner (CEREC Primescan; Dentsply Sirona, Charlotte, NC, USA) to capture the gingival contours and implant position. Then, the scan body was replaced with a reference tooth, and a full-arch scan was performed to support digital abutment design in CAD software (Exocad, Version 3.1 Rijeka; exocad GmbH, Darmstadt, Germany).

**Figure 3:**
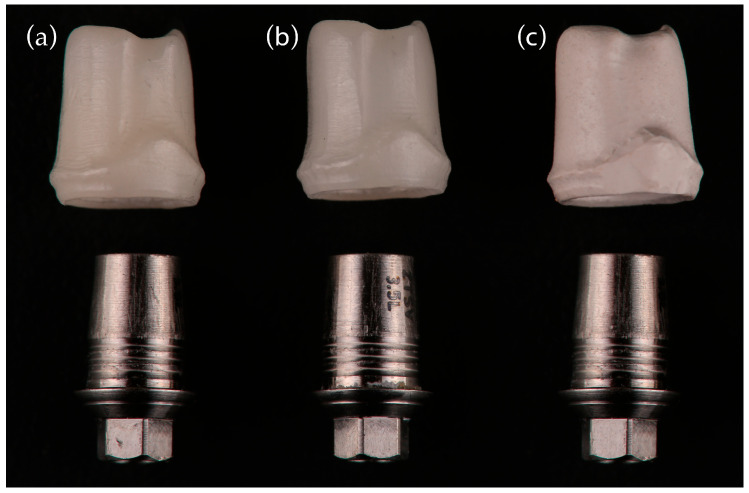
Abutment types used in the study (*n* = 10 each): (**a**) Monolithic zirconia abutment, (**b**) Lithium disilicate abutment, (**c**) BioHPP abutment.

All abutments were customized with a concave emergence profile and standardized to a gingival height of 5 mm, mesiodistal width of 7 mm, buccolingual width of 8 mm, and screw channel diameter of 2.2 mm. Abutments were produced via one-to-one copy milling using a structured-light scanner. All abutments and crowns were designed using CAD software and manufactured under a standardized CAD/CAM workflow. Scanning was performed with a laboratory structured-light scanner (InEos X5; Dentsply Sirona, Bensheim, Germany; accuracy ±5 µm). The CAD designs were processed using hyperDENT CAM software (Version 5.2; FOLLOW-ME! Technology Group, Munich, Germany) with a uniform cement-space setting of 50 µm. Milling was carried out using CAMCube 10 and CAMCube 20 units (imes-icore GmbH, Eiterfeld, Germany) and the CEREC MC XL system (Dentsply Sirona, Bensheim, Germany). Zirconia restorations were sintered in a DEKEMA Austromat 674 furnace (DEKEMA Dental-Keramiköfen GmbH, Freilassing, Germany) at 1450 °C for 2 h; Tessera lithium disilicate crowns were crystallized at 760–820 °C for 4.5 min in the CEREC SpeedFire furnace (Dentsply Sirona, Bensheim, Germany); Cerasmart does not need any sinterization, and BioHPP restorations were cured for 90 s in the bre.Lux PowerUnit 2 (Bredent GmbH & Co. KG, Senden, Germany). All design and fabrication procedures were performed under identical conditions to ensure workflow transparency and reproducibility. Zirconia abutments were fabricated from monolithic multilayer 5Y-TZP blanks (DD CubeX^2^, Dental Direkt GmbH, Spenge, Germany) using a CAMCube M20 unit. Lithium disilicate abutments were milled from IPS e.max CAD blocks (Ivoclar Vivadent AG, Schaan, Liechtenstein) with a CAMCube M10 system. BioHPP abutments were fabricated from ceramic-reinforced PEEK disks (breCAM.BioHPP, Bredent GmbH & Co. KG, Senden, Germany) using the CEREC MC XL system (Dentsply Sirona, Bensheim, Germany). Representative abutment designs are shown in Figure 3.

### 2.4. Crown Fabrication

**Figure 4:**
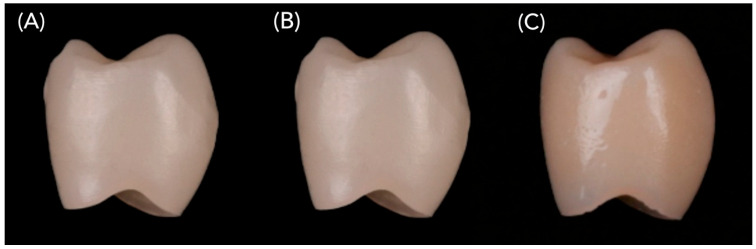
Representative crown types used in the study: (**A**) Monolithic zirconia crown (DD CubeX^2^), (**B**) Advanced lithium disilicate crown (Tessera), (**C**) Hybrid nanoceramic crown (Cerasmart).

Each abutment was restored with a monolithic crown fabricated via a standardized CAD/CAM workflow using Exocad DentalCAD software (Version 3.1 Rijeka; exocad GmbH, Darmstadt, Germany). To ensure morphological uniformity, all crowns were designed with a mesiodistal width of 8.2 mm and an occlusogingival height of 8.8 mm, incorporating a 50 µm cement space. The crowns were milled from one of three materials: monolithic multilayer 5Y-TZP zirconia (DD CubeX^2^; Dental Direkt GmbH, Spenge, Germany), advanced lithium disilicate (CEREC Tessera; Dentsply Sirona, Charlotte, NC, USA), or hybrid nanoceramic (GC Cerasmart; GC Corporation, Tokyo, Japan). Representative examples of these crown types are shown in Figure 4.

The restorations were fabricated using a one-to-one copy-milling workflow. The master crown design was scanned using a structured-light scanner (inEos X5, Dentsply Sirona, Bensheim, Germany) with an accuracy of ≤2 µm. The resulting STL files were processed in Exocad DentalCAD 3.1 Rijeka (Exocad GmbH, Darmstadt, Germany) and then transferred to hyperDENT Compact 9.3 (FOLLOW-ME! Technology Group, Munich, Germany) for toolpath generation. Milling was performed with a five-axis CAMcube M5 unit (B&D Dental Technologies, Salt Lake City, UT, USA) equipped with a four-bur carbide tool set under a standard fine-milling strategy. Additional crowns were produced at Alyans Dental Lab (Izmir, Turkey) using Camcube 10 and Camcube 20 units (EFP Dental GmbH, Haan, Germany) with identical milling parameters to ensure inter-system consistency.

Material-specific post-processing followed manufacturer recommendations. Zirconia crowns underwent stepwise sintering: heating to 900 °C (8 °C/min), ramping to 1450 °C (3 °C/min), holding for 120 min, and cooling to 200 °C (10 °C/min). Tessera crowns were crystallized in a CEREC SpeedFire furnace at 760–820 °C for 4–4.5 min. Cerasmart crowns were milled under identical conditions and required no additional firing. All crowns shared the same geometry, toolpaths, and bur configurations to eliminate fabrication-related variability. Although material-specific surface treatments and resin cements were applied in accordance with manufacturer guidelines, this step introduced a secondary, non-randomized variable associated with bonding-protocol differences.

### 2.5. Hybrid Abutment Assembly

Titanium base components (2 mm height) were air-abraded with 50 µm Al_2_O_3_ at 2 bar pressure for 20 s and subsequently cleaned in an ultrasonic bath with 80% ethanol for 10 min. A thin layer of Ceramic Bond (VOCO GmbH, Cuxhaven, Germany) was applied to dried Ti-bases and allowed to react for 60 s. Zirconia abutments and crowns were sandblasted with 50 µm Al_2_O_3_ at 2 bar pressure for 20 s. Lithium disilicate abutments and crowns were etched with 9% hydrofluoric acid and silanized with Ceramic Bond (VOCO GmbH, Cuxhaven, Germany) for 60 s. Bonding of zirconia and lithium disilicate abutments to Ti-bases was performed using a dual-cure resin cement (Bifix Hybrid Abutment Cement, VOCO GmbH, Cuxhaven, Germany). For BioHPP abutment cementation to Ti-bases, an additional primer (MKZ Primer, Bredent GmbH & Co. KG, Senden, Germany) was applied to the Ti-bases according to the manufacturer’s instructions. For the BioHPP abutments, the bonding protocol involved surface abrasion with 110 µm Al_2_O_3_ at 3 bar, application of Visio.link primer (Bredent GmbH & Co. KG, Senden, Germany), and cementation to titanium bases using DTK-Adhesive resin cement (Bredent GmbH & Co. KG, Senden, Germany), followed by light curing for 90 s.

All abutment assemblies were torqued to the implant analogs at 30 N·cm using a calibrated torque driver. All bonding and cementation procedures were performed strictly according to the manufacturers’ recommendations for each material to achieve optimal adhesion and simulate clinical conditions. Although different surface treatments and cements were used depending on the material type, all specimens followed a standardized workflow consisting of airborne-particle abrasion, ultrasonic cleaning, primer application, and cementation under constant pressure. All procedures were conducted by the same operator under identical environmental conditions to minimize variability and ensure methodological consistency across all groups.

### 2.6. Crown Cementation

**Figure 5:**
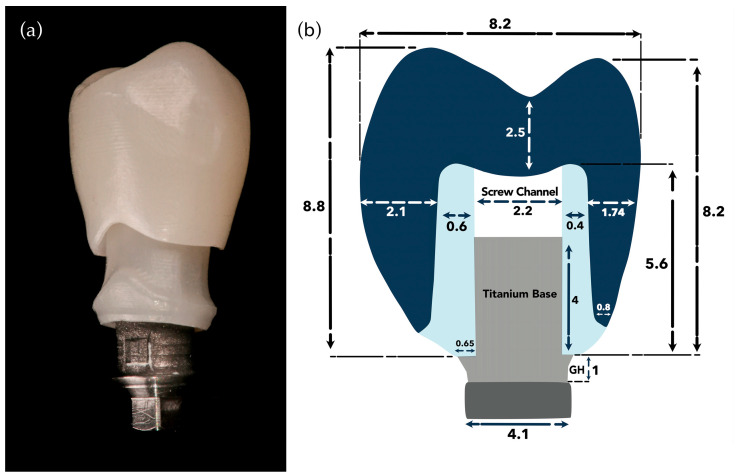
(**a**) Finalized hybrid abutment–crown assembly consisting of a Ti-base, zirconia abutment, and monolithic zirconia crown; (**b**) Schematic representation of the integrated unit with standardized dimensions (in mm).

Crown interiors were treated according to their respective material protocols. Zirconia crowns were sandblasted with 50 µm Al_2_O_3_, ultrasonically cleaned, and bonded with a dual-cure self-adhesive resin cement (Bifix QM, VOCO GmbH, Germany). Tessera crowns were cleaned with 80% ethanol for 10 min, dried, and a thin layer of Ceramic Bond (VOCO GmbH, Cuxhaven, Germany) was applied to the internal surface before cementation. Cerasmart crowns were air-abraded and cemented using a dual-cure self-adhesive resin cement (Bifix QM, VOCO GmbH, Germany) under constant pressure, followed by 45 s of polymerization. For BioHPP abutments, Visio.link primer (Bredent GmbH & Co. KG, Senden, Germany) was applied and light-cured for 90 s before crown placement with DTK-Adhesive resin cement (Bredent GmbH, Germany), which was then polymerized for 180 s. Although different primers and cements were used in accordance with manufacturer protocols, all procedures were standardized to minimize variability. This approach was intentionally adopted to reflect optimized clinical protocols rather than a fully standardized laboratory design. Therefore, the results represent material–cement–primer combinations rather than the isolated effects of abutment or crown materials. Photographic and schematic views of the final restorations are shown in Figure 5.

### 2.7. Thermocycling and Fracture Testing

All specimens were subjected to 5000 thermal cycles between 5 °C and 55 °C, with a dwell time of 20 s, to simulate intraoral aging conditions. This protocol was based on ISO/TS 11405:2015 [17] guidelines, with 5000 cycles approximating one year of clinical service. For fracture testing, the restored implants were secured in stainless-steel holders and subjected to static compressive loading using a universal testing machine (Instron 3345, Instron Corp., Norwood, MA, USA). A 6 mm-diameter spherical stainless-steel loading tip applied load at a 90° angle to the central occlusal fossa of each crown at a crosshead speed of 1 mm/min, up to a maximum load of 5000 N. Failure was defined a priori as catastrophic structural fracture, characterized by visible separation of the restoration. The corresponding load at complete fracture was recorded as the failure load. Fracture loads and testing durations were automatically recorded using Bluehill Lite software (Instron Corp., Norwood, MA, USA). Failure modes, including decementation, cracking, and complete structural fracture, were subsequently examined visually under 10× and 50× magnification using a stereomicroscope (Leica M80, Leica Microsystems GmbH, Wetzlar, Germany).

### 2.8. Statistical Analysis

The normality of data distribution was assessed using the Kolmogorov–Smirnov and Shapiro–Wilk tests. A two-way ANOVA was performed to evaluate the main and interaction effects of abutment and crown materials on fracture strength. Tukey’s honestly significant difference (HSD) test was applied for post hoc multiple comparisons. For each group, mean fracture strength values were reported together with corresponding 95% confidence intervals (CIs). Pairwise comparisons between groups were also accompanied by 95% CIs for mean differences.

The significance level was set at *p* < 0.05. The distribution of fracture modes (crown-only vs. combined abutment–crown) was analyzed using the Chi-square test for independence, with Fisher’s exact test applied where expected cell counts were <5. All statistical analyses, including ANOVA and Chi-square tests, were conducted using SPSS Statistics software (version 22.0; IBM Corp., Armonk, NY, USA).

## 3. Results

### 3.1. Fracture Strength Testing

Fracture strength values varied significantly among the tested groups. All groups exhibited mean fracture loads exceeding 500 N. The ZR–ZR group exhibited the highest mean fracture strength, followed by EM–ZR and BH–ZR. In contrast, the BH–TE group showed the lowest values. Statistically significant differences were found between ZR–ZR and the ZR–TE, ZR–CS, EM–TE, and BH–TE groups (*p* < 0.05). Similarly, EM–ZR demonstrated significantly higher values than ZR–TE, EM–TE, and BH–TE (*p* < 0.05). No statistically significant difference was observed between the EM–TE and BH–TE groups (*p* > 0.05). Descriptive statistics for all groups are presented in Table 2.

**Figure 6:**
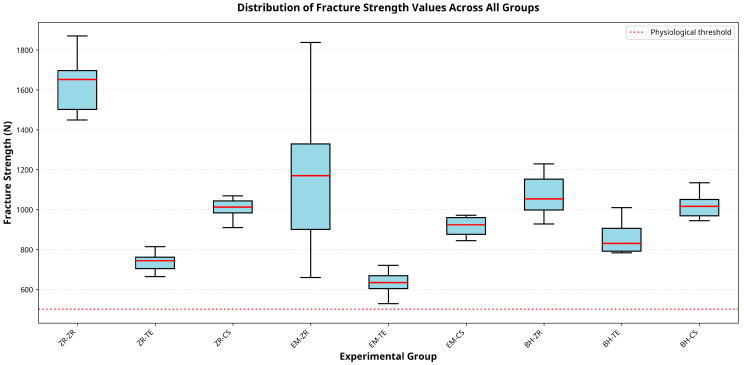
Mean fracture strength values for different material combinations. Mean fracture strength (N) ± standard deviation for nine experimental groups (*n* = 10 per group). Error bars represent standard deviation. Error bars represent standard deviation. Box-and-whisker plots display the distribution of fracture strength values: light blue boxes indicate the interquartile range (25th to 75th percentile), red horizontal lines represent median values, black whiskers extend to minimum and maximum values. The dotted line at 500 N represents the average functional bite force in the premolar region, serving as a clinical threshold for fracture safety. ZR: zirconia; EM: E-max; BH: BioHPP; TE: Tessera; CS: Cerasmart. Statistical differences determined by one-way ANOVA with Tukey HSD post-hoc test (*p* < 0.05).

The differences in fracture strength among the experimental groups are also illustrated in Figure 6, which provides a graphical summary of mean values and associated standard deviations. Different letters indicate statistically significant differences between groups based on Tukey’s HSD test (*p* < 0.05).

### 3.2. Influence of Material Type on Fracture Strength

A two-way ANOVA was conducted to examine the effects of abutment material, crown material, and their interaction on fracture strength. Both main effects were statistically significant: abutment material (F (2,81) = 13.93, *p* < 0.001, η^2^_p_ = 0.256) and crown material (F (2,81) = 101.47, *p* < 0.001, η^2^_p_ = 0.715). Crown material demonstrated a large effect size (ω^2^ = 0.574), explaining approximately 57% of the variance in fracture strength (Table 3).

The interaction between abutment and crown materials was also significant (F (4,81) = 9.36, *p* < 0.001, η^2^_p_ = 0.316), indicating that the effect of abutment material depended on the crown material used. Levene’s test indicated heterogeneity of variances (*p* < 0.001), which was addressed through robust interpretation and effect-size reporting.

### 3.3. Interaction Between Abutment and Crown Materials

**Figure 7:**
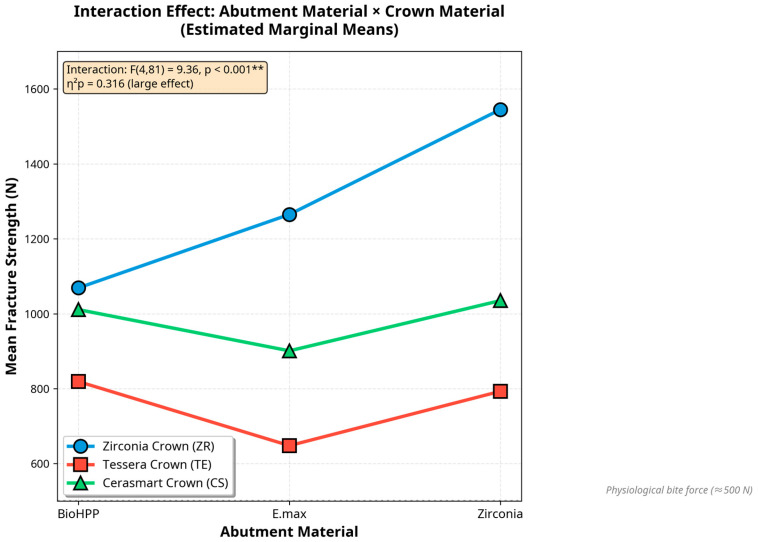
Interaction effect between abutment and crown materials on fracture strength (estimated marginal means with 95% CI). The significant interaction (F (4,81) = 9.36, ** *p* < 0.001, η^2^_p_ = 0.316) shows that fracture strength depends on both material types, with zirconia crowns achieving the highest values (ZR–ZR: 1545 N). All groups exceeded the physiological bite force threshold (≈500 N).

The interaction effect showed that fracture strength varied according to specific material combinations. Zirconia crowns achieved the highest fracture strength overall, particularly when paired with zirconia abutments. All groups exceeded the clinical bite force threshold (≈500 N) (Figure 7).

### 3.4. Failure Mode Analysis

The distribution of failure modes (crown-only vs. combined abutment–crown) differed significantly among the experimental groups (χ^2^ (8) = 16.3, *p* = 0.039). Post hoc analysis revealed that abutment material had a highly significant effect (χ^2^ (2) = 11.3, *p* = 0.004), with BioHPP abutments showing exclusively crown-only fractures. In contrast, zirconia (ZR) and lithium disilicate (EM) abutments exhibited a mix of crown-only and combined abutment–crown fractures. Crown material did not significantly influence failure mode (χ^2^ (2) = 1.67, *p* = 0.435). Cramér’s V indicated moderate to large effect sizes (overall: V = 0.425; by abutment: V = 0.354) for overall and abutment-specific comparisons (Table 4).

**Figure 8:**
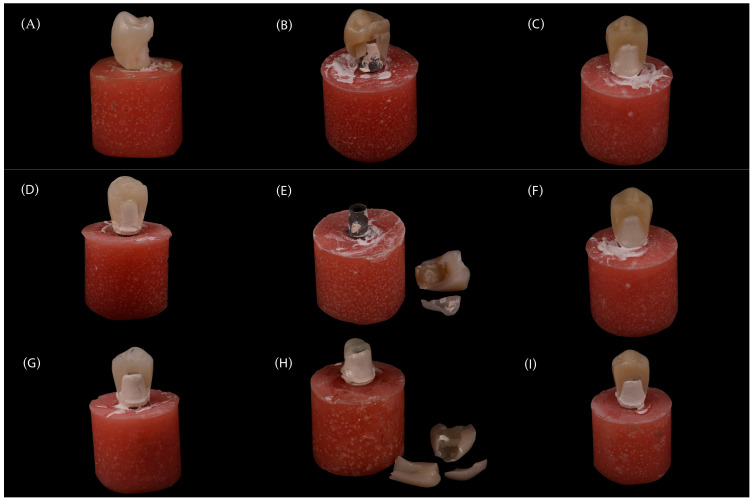
Post-fracture images of tested specimens: (**A**) ZR–ZR, (**B**) ZR–TE, (**C**) ZR–CS, (**D**) EM–ZR, (**E**) EM–TE, (**F**) EM–CS, (**G**) BH–ZR, (**H**) BH–TE, and (**I**) BH–CS. No fractures involving the screw, implant platform, or titanium base were detected in any group. Abbreviations: ZR, zirconia; EM, lithium disilicate; BH, BioHPP; TE, Tessera; CS, Cerasmart.

Representative post-fracture images from each group are shown in Figure 8, illustrating the characteristic failure patterns observed during mechanical testing. No fractures involving the screw, implant platform, or titanium base occurred, emphasizing the protective role of the abutment material in dissipating stress during loading.

Detailed group-wise failure distributions are presented in Table 5, confirming that all BioHPP (BH) abutment groups (*n* = 30) exhibited exclusively crown-only fractures, whereas ZR and EM abutments showed varying proportions of combined failures.

**Figure 9:**
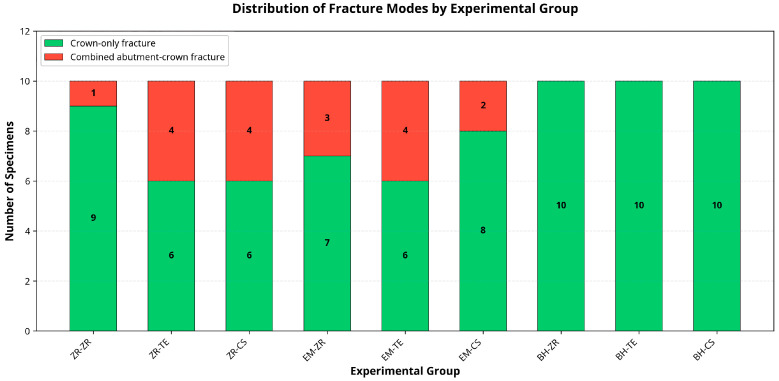
Distribution of fracture modes by experimental group. BioHPP abutments (*n* = 30) exhibited exclusively crown-only fractures, differing significantly from zirconia and lithium disilicate abutments, which showed mixed failure patterns (χ^2^ (2) = 11.3, *p* = 0.004, Fisher’s exact *p* < 0.001).

The distribution of fracture types is summarized in Figure 9, showing that all BioHPP abutments failed exclusively at the crown level, whereas zirconia and lithium disilicate abutments displayed both crown-only and combined fractures. This pattern reflects a more favorable, energy-absorbing failure behavior for polymer-based abutments.

## 4. Discussion

The present in vitro study evaluated the mechanical behavior of metal-free CAD/CAM restorative systems by combining three different abutment materials (zirconia, lithium disilicate, and BioHPP) with three types of crowns, all bonded to prefabricated titanium bases. This two-piece hybrid design, involving a titanium base and a bonded ceramic or polymer component, was selected to reflect current clinical trends and ensure standardized testing. The null hypothesis stated that neither the abutment material nor the crown material, nor their interaction, would significantly affect the fracture strength of the tested restorations; however, based on the observed differences among the groups, this hypothesis was rejected.

Previous studies, including Edelhoff et al., have shown that two-piece hybrid abutments supported by titanium bases provide improved marginal fit and mechanical stability compared to one-piece ceramic designs [12]. This is primarily due to the stress-distributing function of the titanium base. In the present study, zirconia abutments combined with titanium bases demonstrated the highest resistance to fracture among all tested configurations, supporting previous findings and emphasizing the mechanical benefits of the two-piece implant restoration design.

The ZR–ZR group demonstrated the highest fracture strength (1417 N), supporting previous findings by Favásuli et al., who concluded that zirconia hybrid abutments, especially those supported by titanium bases, offer greater resistance to mechanical failure compared to other ceramic options [23].

Takano et al. evaluated implant-supported hybrid abutment crowns fabricated from 4Y-partially stabilized zirconia (4Y-PSZ) and reported a mean fracture load of approximately 2060 N, which was higher than the 1417 N obtained for 5Y-TZP zirconia in the present study [21]. This variation may be attributed to differences in the composition and processing of the zirconia materials. It is well established that as the yttria content in zirconia increases from 3Y to 5Y, translucency improves while flexural strength decreases. The higher tetragonal phase fraction in 4Y-PSZ enhances transformation toughening and crack resistance, whereas the greater cubic phase content in 5Y-TZP improves translucency but reduces mechanical strength. In this study, 5Y-TZP was intentionally selected for both abutment and crown components to reflect contemporary clinical trends prioritizing improved esthetics in posterior regions while maintaining adequate functional strength.

However, these findings should be interpreted cautiously, as the present investigation was conducted under static in vitro conditions without cyclic fatigue loading, which limits direct extrapolation to long-term clinical performance.

Despite laboratory concerns, zirconia abutments have shown promising clinical outcomes. Sailer et al. reported a 5-year survival rate of 99.1% for ceramic abutments, like titanium, along with fewer technical complications (6.9% vs. 15.9%) [24]. While short follow-up durations and protocol heterogeneity were noted limitations in their systematic review, these findings support the potential long-term clinical reliability of zirconia abutments, particularly in anterior regions.

Several clinical studies have supported the promising performance of tooth-colored abutments in implant restorations. According to a meta-analysis by Cao and colleagues, zirconia abutments demonstrated clinical performance metrics like those of their titanium counterparts, emphasizing their viability in clinical practice [25]. Similarly, Laumbacher et al. demonstrated that zirconia abutments restored with lithium disilicate crowns exhibited high survival (98%) and low technical complication rates over a 5-year period, reinforcing the clinical reliability of all-ceramic solutions [26].

BioHPP abutments combined with hybrid ceramics or lithium disilicate crowns demonstrated lower strength than zirconia-based combinations. This finding is in line with previous work by Elsayed et al., where BioHPP-based assemblies were less fracture-resistant [27].

Contrary to Fayed et al., who found no statistically significant difference between Tessera and e.max CAD (*p* = 0.053), Tessera crowns in our study exhibited the lowest mean fracture resistance (823 N) [28]. These differences may be attributed to varying testing protocols, aging conditions, and cementation techniques. Although Tessera offers fast crystallization and optical benefits, its mechanical limitations under high-load conditions may restrict its use in posterior regions.

The findings from El-Mahdy et al. indicated that polymer-based abutments can reduce peri-implant stress [29]. In our study, the BH–CS group (1118 N) performed better than PEKK–composite combinations previously reported (351 N), likely due to the superior strength of hybrid ceramics.

Additionally, the biomimetic framework suggested by Tribst et al. proposes pairing a low-modulus abutment (dentin analog) with a high-modulus crown (enamel analog) to improve fracture behavior [30]. Our results support this approach: BH–ZR yielded higher resistance (1138 N) than BH–TE (823 N), though modulus compatibility alone was insufficient; material strength remained a decisive factor.

All recorded fracture loads for lithium disilicate abutments exceeded the average functional bite forces for the premolar region (400–500 N), indicating a substantial safety margin under physiological conditions [30]. However, this value represents a general reference range rather than a clinical failure threshold, as individual bite forces can vary widely. Therefore, these findings should be interpreted only as a contextual comparison under static loading conditions, not as direct evidence of clinical durability or safety margins.

Notably, no fractures involved the titanium base, abutment screw, or implant platform in any group, supporting existing data showing that screw fractures in single-unit restorations are rare (0.35–0.6%) [31]. Most failures occurred in the crown or at the abutment–crown interface, consistent with previous reports emphasizing that such failures are more clinically manageable than abutment or screw fractures [10,19,32,33].

A key finding of this study is the significant difference in failure modes, where BioHPP abutments exclusively led to more reparable, crown-only fractures. The observed statistical significance (*p* = 0.004) indicates that the reduced rigidity of high-performance polymers might help in dissipating applied stresses, protecting the underlying abutment and implant connection from catastrophic failure. This is a critical clinical consideration, as crown-only fractures are generally manageable with repair or replacement, whereas abutment-level failures often necessitate more complex and costly interventions.

From a clinical standpoint, although all tested groups exceeded normal bite force values for premolars, these findings should be interpreted cautiously since static loading does not represent long-term intraoral performance. While static fracture testing cannot reproduce the complex cyclic and multidirectional forces present in the oral environment, it remains an essential first step in the mechanical evaluation of restorative materials. Static load-to-fracture testing provides a standardized and reproducible assessment of a material’s ultimate strength under a worst-case single-event overload, allowing meaningful baseline comparisons across material combinations. These initial results help identify materials with adequate structural resilience before they are subjected to more demanding fatigue protocols and contribute to predicting which restorations are more likely to withstand occlusal forces in clinical scenarios. Differences exceeding these thresholds may not translate into improved clinical outcomes, as fracture behavior, reparability, and esthetic management are often more relevant in daily practice. Therefore, clinicians should not only consider high fracture strength but also favor material combinations likely to fail in more reparable modes under stress.

This study was conducted in vitro using static loading without dynamic mechanical fatigue, which may not replicate long-term clinical conditions. A further limitation is the absence of fractographic analysis, such as scanning electron microscopy (SEM), to characterize the fracture surfaces. SEM evaluation could have identified fracture origins, crack initiation sites, and propagation pathways, providing deeper insight into the underlying failure mechanisms of the different material combinations. Future investigations incorporating SEM-based fractography would therefore offer a more comprehensive understanding of how these materials behave under functional loading. Thermocycling (5000 cycles) was used to simulate aging, but this represents less than one year of intraoral service. Another limitation of this study is the use of different bonding and surface conditioning protocols based on material-specific manufacturer recommendations. While clinically realistic, this heterogeneity prevents isolating the pure effect of the restorative material on fracture strength. Because different resin-based luting agents and surface treatments were used according to each material’s manufacturer protocol, the findings should be interpreted as combination-dependent rather than solely material-dependent. Although this approach enhances clinical relevance, it may also introduce systematic variability in bonding performance, which could influence stress distribution and failure modes.

Further research should involve cyclic loading protocols, long-term clinical studies, and standardized bonding protocols to better evaluate the durability of hybrid abutment–crown systems.

## 5. Conclusions

- Zirconia-based restorations demonstrated the highest fracture resistance, making them suitable for posterior regions with high functional demands.
- For premolar regions where esthetics are a priority, more translucent materials like advanced lithium disilicate and hybrid ceramics can be considered, despite their lower fracture resistance.
- A balanced approach considering strength, esthetics, and reparability is crucial for material selection.
- Further long-term clinical studies are necessary to validate these in vitro findings.

## Figures and Tables

**Table 1:** Experimental groups showing the combinations of abutment and crown materials (*n* = 10 per group), and the corresponding group codes.

Group	*n*	Abutment Material	Crown Material	Code
A	10	Monolithic zirconia (ZR)	Monolithic zirconia (ZR)	ZR–ZR
B	10	Monolithic zirconia (ZR)	Advanced Lithium disilicate (Tessera, TE)	ZR–TE
C	10	Monolithic zirconia (ZR)	Hybrid ceramic (Cerasmart, CS)	ZR–CS
D	10	Lithium disilicate (EM)	Monolithic zirconia (ZR)	EM–ZR
E	10	Lithium disilicate (EM)	Advanced Lithium disilicate (Tessera, TE)	EM–TE
F	10	Lithium disilicate (EM)	Hybrid ceramic (Cerasmart, CS)	EM–CS
G	10	Ceramic-reinforced polymer (BH)	Monolithic zirconia (ZR)	BH–ZR
H	10	Ceramic-reinforced polymer (BH)	Advanced Lithium disilicate (Tessera, TE)	BH–TE
I	10	Ceramic-reinforced polymer (BH)	Hybrid ceramic (Cerasmart, CS)	BH–CS

**Table 2:** Descriptive statistics of all specimens included in the study.

	*n*	$\bar{x}$	SD ±	Min	Max	Se	Skew
ZR–ZR *	10	1417.22	285.39	1095.29	1989.03	90.25	0.935
ZR–TE	10	864.01	133.54	622.10	1113.50	42.23	−0.040
ZR–CS	10	1059.04	236.02	710.30	1302.83	74.64	−0.332
EM–ZR *	10	1348.67	269.45	1107.06	1842.61	85.21	1.030
EM–TE	10	947.56	123.18	762.93	1167.63	38.95	0.217
EM–CS	10	1128.83	185.02	932.66	1522.79	58.51	1.180
BH–ZR	10	1137.97	122.20	894.76	1280.56	38.64	−1.105
BH–TE *	10	823.45	75.48	665.15	913.23	23.87	−0.851
BH–CS	10	1118.15	125.80	939.46	1341.56	39.78	0.291

Note: Data are presented as mean ± standard deviation. SE = standard error of the mean. All groups showed acceptable normality (Shapiro–Wilk *p* > 0.05 for most groups) and were analyzed using parametric tests. * *p* < 0.05. Abbreviations: $\bar{x}$, mean; SD, standard deviation; Min, minimum; Max, maximum; SE, standard error; Skew, skewness.

**Table 3:** Two-Way ANOVA Results for Fracture Strength.

Source	df	Sum of Squares	Mean Square	F	*p*	η^2^_p_	ω^2^
Abutment Material	2	604,194	302,097	13.93	<0.001	0.256	0.074
Crown Material	2	4,400,000	2,200,000	101.47	<0.001	0.715	0.574
Abutment × Crown	4	812,232	203,058	9.36	<0.001	0.316	0.096
Residuals	81	1,760,000	21,685	—	—	—	—
Total	89	7,576,426	—	—	—	—	—

E Effect sizes: η^2^_p_ = partial eta-squared (effect size indicating the proportion of variance explained by a factor, excluding other factors); ω^2^ = omega-squared (adjusted effect size estimating the proportion of variance explained in the population, less biased than η^2^_p_). df = degrees of freedom; F = F-statistic; *p* = probability value.

**Table 4:** Chi-Square Analysis of Failure Mode Distribution.

Analysis	χ^2^	df	*p*-Value	Fisher’s Exact *p*	Cramér’s V	Interpretation
Overall (9 groups)	16.3	8	0.039	0.021	0.425	Significant association
By Abutment Material	11.3	2	0.004	<0.001	0.354	Highly significant
By Crown Material	1.67	2	0.435	0.488	0.136	Not significant

**Table 5:** Detailed Failure Mode Distribution by Experimental Group.

Group	Crown-Only *n* (%)	Combined *n* (%)	Total
ZR–ZR	9 (90.0%)	1 (10.0%)	10
ZR–TE	6 (60.0%)	4 (40.0%)	10
ZR–CS	6 (60.0%)	4 (40.0%)	10
EM–ZR	7 (70.0%)	3 (30.0%)	10
EM–TE	6 (60.0%)	4 (40.0%)	10
EM–CS	8 (80.0%)	2 (20.0%)	10
BH–ZR	10 (100%)	0 (0.0%)	10
BH–TE	10 (100%)	0 (0.0%)	10
BH–CS	10 (100%)	0 (0.0%)	10
Total	72 (80.0%)	18 (20.0%)	90

Abbreviations: ZR, zirconia; EM, lithium disilicate; BH, BioHPP; TE, Tessera; CS, Cerasmart.

## Data Availability

The raw data supporting the conclusions of this article will be made available by the authors on request.

## Abbreviations

The following abbreviations are used in this manuscript:

3Y-TZP	3 mol% Yttria-stabilized Tetragonal Zirconia Polycrystal
4Y-PSZ	4 mol% Yttria-partially Stabilized Zirconia
5Y-TZP	5 mol% Yttria-stabilized Tetragonal Zirconia Polycrystal
BH	BioHPP (Biocompatible High-Performance Polymer)
CAD/CAM	Computer-Aided Design/Computer-Aided Manufacturing
CS	Cerasmart (Hybrid Nanoceramic)
EM	IPS e.max (Lithium Disilicate)
FDP	Fixed Dental Prostheses
HACs	Hybrid Abutment Crowns
HSD	Honestly Significant Difference
PEEK	Polyetheretherketone
TE	Tessera (Advanced Lithium Disilicate)
Ti	Titanium
Ti-base	Titanium Base
ZR	Zirconia
